# Association between tobacco use, including heated tobacco products, and problem gambling: A cross-sectional study

**DOI:** 10.1016/j.abrep.2026.100678

**Published:** 2026-02-21

**Authors:** Minami Takeda, Takashi Yoshioka, Kosuke Shido, Atsushi Hozawa, Takahiro Tabuchi

**Affiliations:** a School of Medicine, Tohoku University, Miyagi, Japan; b Institute of Clinical Epidemiology, Showa Medical University, Tokyo, Japan; c Health Technology Assessment Unit, Department of Preventive Medicine and Public Health, Keio University School of Medicine, Tokyo, Japan; d Department of Dermatology, Tohoku University Graduate School of Medicine, Miyagi, Japan; e Division of Epidemiology, School of Public Health, Tohoku University Graduate School of Medicine, Miyagi, Japan

**Keywords:** Problem gambling, Smoking, Heated tobacco products, Public health, Risk factor, Epidemiology

## Abstract

**Background:**

Although smoking is a known risk factor for problem gambling, the relationship between heated tobacco products (HTPs), emerging tobacco products that are widely used in Japan, and problem gambling has not yet been examined. This study investigated the association between smoking behaviour, including HTP use, and problem gambling.

**Methods:**

We conducted a cross-sectional study using data from a large-scale online survey conducted from January 24 to February 27, 2024. Respondents who reported gambling within the past year were included in this study. Exclusive cigarette use, exclusive HTP use, and dual use were defined as smoking variables based on current smoking patterns in Japan. Problem gambling was defined as a score of 8 or higher on the Problem Gambling Severity Index. Multivariable weighted logistic regression models were fitted to examine the adjusted odds ratios (AORs) of smoking status for problem gambling.

**Findings:**

In total, 12,222 respondents (7,649 men and 4,573 women) were included in the study. The proportion of problem gambling differed; it was 4.6% among individuals who did not use tobacco products, 13.8% among those who exclusively used combustible cigarettes, 15.2% among those who exclusively used HTP, and 41.9% among those who used both. Compared to non-use/non-current use of tobacco products, exclusive combustible cigarette use (AOR, 2.89 [95% CI, 2.36–3.53]), exclusive HTP use (AOR, 2.34 [95% CI, 1.81–2.99]), and dual use (AOR, 10.52 [95% CI, 8.89–12.47]) were associated with problem gambling.

**Conclusions:**

Smoking behaviours, including heated tobacco product use, were associated with problem gambling.

## Highlights

• First study examining heated tobacco products (HTPs) and problem gambling link.
• 12,222 Japanese people who gambled surveyed; tobacco users and non-users analyzed.
• All tobacco use patterns were associated with PG.
• Dual use of cigarettes and HTPs showed notably high odds ratios for PG.
• Women and younger adults showed clear tobacco-PG associations.

## 1 Introduction

Problem gambling, characterised by difficulties in limiting money and/or time spent gambling, has adverse consequences for individuals who gamble, others, and the community (University of Adelaide, 2005). The prevalence of problem gambling in the general public is approximately 1.29% globally and 1.7% in Japan (Gabellini et al., 2022, Hwang et al., 2024). Although the prevalence of problem gambling is only approximately 1–2%, dealing with it is important because of its huge negative impact on society. For example, in England, problem gambling is estimated to have an annual overall societal cost of between ₤1.05 billion and ₤1.77 billion (approximately $1.43 billion to $2.42 billion) (Office for Health Improvement & Disparities, 2023). However, problem gambling is a reversible condition, and it is important to tailor interventions to the underlying motivations of each individual (Milosevic and Ledgerwood, 2010, Neighbors et al., 2002). Therefore, identifying factors associated with problem gambling is essential for providing effective treatment.

Tobacco use is a well-established factor associated with problem gambling. Several studies have shown that the co-occurrence rates of problem gambling and tobacco use or nicotine dependence range from 41% to 60% (Bui et al., 2023, Håkansson and Ek, 2018, Lorains et al., 2011). Furthermore, it has been suggested that individuals who engage in both problem gambling and smoking have more severe gambling disorders (Grant et al., 2008, McGrath et al., 2012). One reason many individuals who smoke engage in problem gambling is that individuals who smoke tend to have a strong motivation to obtain rewards and alleviate pain (McGrath et al., 2012). In a study focusing on the risk of relapse after treatment for problem gambling, it was reported that among individuals who presented with severe problem gambling, individuals who used tobacco daily had a significantly higher relapse rate after treatment than those who did not (Bui et al., 2023). Thus, the associations between problem gambling and tobacco are important for identifying the motivations behind problem gambling and effective treatment.

Heated tobacco products (HTPs), which are new tobacco products, are potentially related to problem gambling, similar to combustible cigarettes. Between 2015 and 2020, HTP usage increased from 0.12% to 10.57% in Western Pacific countries, and from 0% to 1.5% in European countries (Sun et al., 2023). The prevalence of HTP use in Japan, where sales of electronic cigarettes (e-cigarettes), including nicotine, are prohibited, is high, at 12.4% in 2023 (Odani & Tabuchi, 2024). However, to date, no study has examined the relationship between HTP use and problem gambling. With the rapid proliferation of HTPs, smoking patterns are expected to become increasingly diversified. For example, patterns may include not only the exclusive use of HTPs, but also the concurrent use of HTP alongside combustible cigarettes. Therefore, it is essential to consider diverse smoking patterns when examining the relationship between HTPs and problem gambling. We hypothesized that smoking remains associated with problem gambling even in countries where HTP use is widespread, such as Japan. In this context, we examined the association between problem gambling and tobacco use, including only HTPs use and dual use of HTPs and combustible cigarettes in Japan.

## 2 Methods

### 2.1 Data

This study used a cross-sectional design to analyse data from a comprehensive online survey in Japan. This nationwide survey was conducted online and relied on self-reported data. The selection process accounted for various demographic and socioeconomic factors, including smoking, education, housing, spouse, region, and self-rated health status, based on definitions from the Japan census (Tabuchi et al., 2019). The survey was conducted between January 24 and February 27, 2024.

### 2.2 Inclusion and exclusion criteria

This study included respondents who had engaged in gambling activities in the past year. Respondents with implausible responses were excluded to ensure data integrity. Examples of such responses included significantly shorter response times, consistent selection of the same answer across multiple questions, choosing all options for questions regarding illegal substance use or chronic conditions, and failing attention checks.

### 2.3 Variables

#### 2.3.1 Exposure: prevalence of current tobacco product use

The prevalence of current tobacco product use was determined based on the self-reported use of combustible cigarettes, HTPs, and the dual use of combustible cigarettes and HTPs within the past 30 days. Participants who reported using any tobacco product on one or more days in the past 30 days were classified as individuals who currently used. The survey included a dedicated section on combustible cigarettes and a detailed list of HTP brands, such as Ploom Tech, Ploom X, IQOS, glo, and lil HYBRID, to enhance clarity and ensure accurate responses. These brand-specific questions were designed to improve the response accuracy and were considered highly valid for identifying individuals who currently used tobacco product (Odani & Tabuchi, 2022).

#### 2.3.2 Outcome: gambling engagement and problem gambling

Participants were surveyed regarding their engagement in 10 types of gambling activities: horse racing, bicycle racing, boat racing, auto racing, casino, lottery, sports promotion lottery, pachinko/slot, stock/commodities or foreign exchange trading, and cryptocurrency trading. Respondents were asked to indicate the frequency of their participation using the following categories: “never”, “within the past year (less than once a week)”, “within the past year (once a week or more)”, “over a year ago (less than once a week)”, and “over a year ago (once a week or more)”. Questionnaires and response options followed the South Oaks Gambling Screen (SOGS) (Kido & Shimazaki, 2007). Individuals who reported engaging in any of these activities within the past year, regardless of the frequency, were classified as those who had gambled recently.

Gambling severity was assessed using the Problem Gambling Severity Index (PGSI) (Ferris et al., 2001), a 9-item tool developed to identify problem gambling in the general population. The Japanese version of the PGSI has been previously validated (So et al., 2019). According to the original framework of the PGSI (Ferris et al., 2001), a score of 0 indicates no gambling problems, whereas scores of 1–2, 3–7, and 8–27 correspond to low-risk, moderate-risk, and problem-gambling categories, respectively. Consistent with a prior study (Yoshioka et al., 2025), we adopted this classification and defined problem gambling as a PGSI score of 8 or higher.

#### 2.3.3 Covariates

A broad set of covariates, which could be associated with problematic gambling behaviour, included (1) sex (Volberg et al., 2018, Williams et al., 2021), (2) age (emerging adulthood, 16–29 years; young and middle adulthood, 30–45 years; middle to late adulthood, 46–65 years; and post-retirement, 66–83 years) (Halloran, 2024, Volberg et al., 2018, Xu et al., 2025), (3) education (high school graduate and less/college graduate or more) (Volberg et al., 2018, Xu et al., 2025), (4) marital status (never married, married, or divorced/widowed) (Valenciano-Mendoza et al., 2023), (5) self-rated health (excellent/very good/good or fair/poor) (Volberg et al., 2018), (6) alcohol consumption (never/non-current or current) (Sharman et al., 2019, Volberg et al., 2018), and (7) equivalent household income (1st quartile, 2nd quartile, 3rd quartile, 4th quartile, or unknown/declined to answer) (Volberg et al., 2018, Williams et al., 2021). Equivalent household income was calculated by dividing household income by the square root of household size (Fukuda et al., 2007).

### 2.4 Statistical analyses

First, the number and percentage of respondents within each sociodemographic category and the proportion of individuals with problem gambling were described. Based on tobacco use in the last 30 days, respondents were divided into four groups: individuals who did not use tobacco products, those who exclusively used combustible cigarettes, those who exclusively used HTPs, and those who used both combustible cigarettes and HTPs. Next, univariate comparisons of each variable across the tobacco use categories were performed using the chi-square test. Finally, a weighted logistic regression model was fitted to estimate the adjusted odds ratios (AORs) of problem gambling and the corresponding 95% confidence intervals (CIs). Marital status and the presence or absence of equivalent household income information were treated as dummy variables.

In this study, we assumed heterogeneity by sex and age and conducted subgroup analyses based on these factors (Volberg et al., 2018, Williams et al., 2021). For each subgroup, a regression model adjusted for the same covariates used in the primary analysis was fitted.

All analyses were weighted using inverse probability weighting (IPW) to address the potential selection bias inherent in the Internet-based sample. To derive IPW, a propensity score for “participation in the Internet survey” was calculated using logistic regression, incorporating data from the 2024 JASTIS sample and a nationally representative sample (Ministry of Health, Labour and Welfare, 2023, Tabuchi et al., 2019). This adjustment accounted for demographic, socioeconomic, health-related, and tobacco-use-related factors (Tabuchi et al., 2019). Details of the weighting methodology are available in literature (Schonlau et al., 2009). Statistical significance was set at p < 0.05. All statistical analyses were performed using R version 4.4.2.

### 2.5 Ethical considerations

All respondents completed online questionnaires after providing digital informed consent, indicating their willingness to participate in the survey. Institutional ethical approval for this study was granted by Osaka International cancer Institute (approval number: 1611079163–3) and Tohoku University Graduate School of Medicine (approval number: 2024–1-231).

## 3 Results

**Fig. 1:**
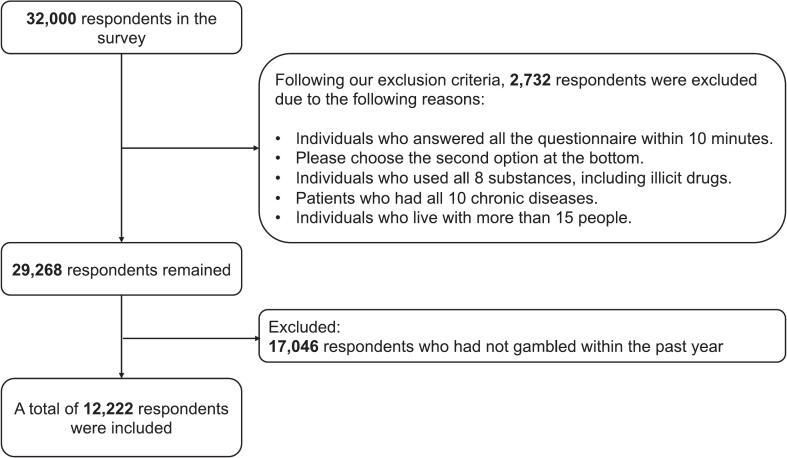
Flow diagram of this study.

A total of 12,222 individuals were eligible for inclusion in this study (Fig. 1). Table 1 presents the characteristics of the included respondents. Based on PGSI scores, 10.9% were classified as individuals who presented problem gambling (PGSI ≥ 8), with a prevalence of 4.6% among individuals who did not use tobacco products, 13.8% among those who exclusively used combustible cigarettes, 15.2% among those who exclusively used HTPs, and 41.9% among those who used both, respectively. Regarding demographic characteristics, 36.9% were women and 44.9% were aged 16–45 years. Furthermore, 43.6% and had completed college graduation or above and 63.0% were married. Regarding health-related factors, 86.9% answered good as self-rated health, whereas 60.9% had consumed alcohol within a month. All factors presented statistically significant differences in the univariate comparisons (p < 0.001).

**Table 1:** Baseline characteristics of respondents.

	Overall	Non-current/never use	Exclusive cigarette use	Exclusive HTP use	Dual use	p-value*
	n (%)	n (%)	n (%)	n (%)	n (%)	
Overall	12,222	8439	1550	769	1464	
**PGSI score**						
Non-problem gambling (0–7 points)	10,837 (89.1)	8049 (95.4)	1330 (86.2)	656 (84.8)	802 (58.1)	<0.001
**Problem gambling (8**–**27 points)**	**1385 (10.9)**	**390 (4.6)**	**220 (13.8)**	**113 (15.2)**	**662 (41.9)**	

**Sociodemographic characteristics**						
**Sex**						
Female	4573 (36.9)	3707 (44.6)	358 (20.0)	203 (24.0)	305 (18.9)	<0.001
Male	7649 (63.1)	4732 (55.4)	1192 (80.0)	566 (76.0)	1159 (81.1)	
**Age (years)**						
16–29	2271 (17.4)	1459 (16.9)	166 (8.5)	141 (17.1)	505 (30.5)	<0.001
30–45	3346 (27.5)	2263 (25.2)	362 (26.4)	237 (37.2)	484 (36.7)	
46–65	4163 (34.5)	2793 (33.4)	657 (44.6)	331 (40.4)	382 (25.8)	
66–83	2442 (20.5)	1924 (24.4)	365 (20.5)	60 (5.3)	93 (6.9)	
**Education**						
High school graduate and less	3458 (56.4)	3972 (73.9)	818 (78.2)	390 (78.0)	586 (68.5)	<0.001
College graduate or more	8764 (43.6)	4467 (26.1)	732 (21.8)	379 (22.0)	878 (31.5)	
**Marital status**						
Never married	3935 (27.6)	2681 (27.0)	460 (24.4)	219 (25.7)	575 (35.5)	<0.001
Married	7242 (63.0)	5070 (63.0)	913 (66.0)	468 (64.7)	791 (58.3)	
Divorced/Widowed	1045 (9.4)	688 (9.9)	177 (9.6)	82 (9.6)	98 (6.2)	
**Self-rated health**						
Excellent/very good/good	10,086 (86.9)	7072 (87.3)	1242 (85.9)	626 (86.7)	1146 (85.6)	<0.001
Fair/poor	2136 (13.1)	1367 (12.7)	308 (14.1)	143 (13.3)	318 (14.4)	
**Alcohol consumption**						
Never/non-current	4433 (39.1)	3477 (45.2)	411 (28.1)	232 (30.1)	313 (21.8)	<0.001
Current	7789 (60.9)	4962 (54.8)	1139 (71.9)	537 (69.9)	1151 (78.2)	
**Equivalent household income**						
1st quartile	2795 (26.0)	1961 (27.1)	375 (26.3)	126 (16.6)	333 (24.8)	<0.001
2nd quartile	2353 (19.5)	1575 (18.2)	306 (18.5)	157 (24.3)	315 (25.2)	
3rd quartile	2239 (17.8)	1501 (16.9)	272 (18.6)	175 (23.7)	291 (18.6)	
4th quartile	2665 (16.6)	1762 (15.7)	335 (17.5)	201 (18.5)	367 (19.9)	
Unknown/declined to answer	2170 (20.1)	1640 (22.1)	262 (19.1)	110 (16.8)	158 (11.4)	

Abbreviations: HTP, heated tobacco product; PGSI, Problem Gambling Severity Index.

* Based on chi-squared test.

Table 2 presents the prevalence and AOR of problem gambling in each tobacco use category. Tobacco product use was associated with the presence of problem gambling (exclusive combustible cigarette use, AOR = 2.89; 95% CI = 2.36–3.53, p < 0.001; exclusive HTP use, AOR = 2.34; 95% CI = 1.81–2.99, p < 0.001; and dual use, AOR = 10.52; 95% CI = 8.89–12.47, p < 0.001).

**Table 2:** Comparison of the prevalence and adjusted odds ratios of problem gambling by smoking status.

	Problem gambling, weighted %	AOR (95% CI)*	p-value
Non-current/never use	4.6	1.00 (ref.)	(ref.)
Exclusive cigarette use	13.8	**2.89 (2.36–3.53)**	**<0.001**
Exclusive HTP use	15.2	**2.34 (1.81–2.99)**	**<0.001**
Dual use	41.9	**10.52 (8.89–12.47)**	**<0.001**

Abbreviations: AOR, adjusted odds ratio; HTP, heated tobacco product; 95% CI, 95% confidence interval.

*Adjusted for sex, age group, education, marital status, self-rated health, alcohol consumption, and equivalent household income.

**Fig. 2:**
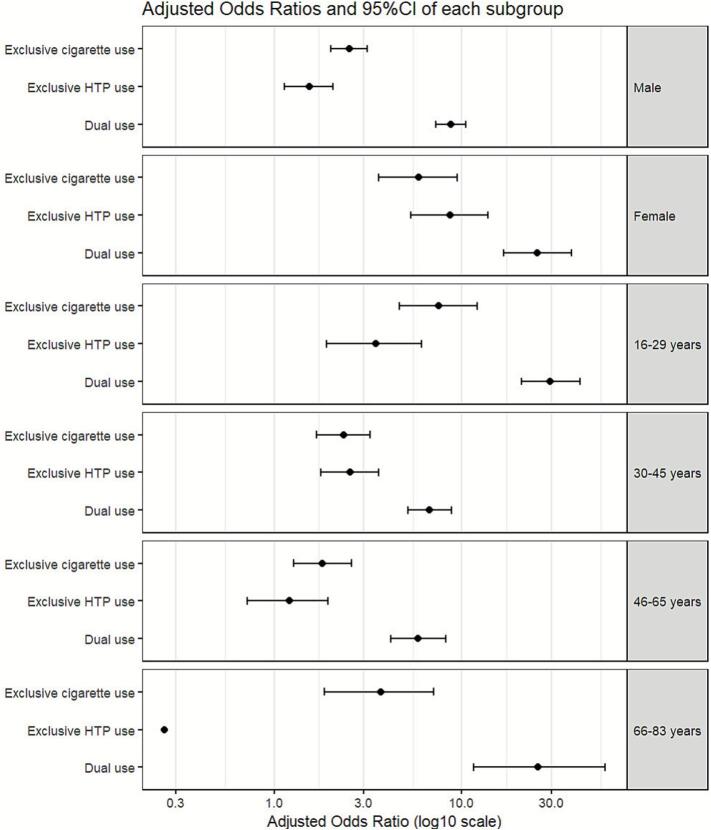
Prevalence and odds ratios of problem gambling by tobacco use status for each sex and age groups. **Note.** Due to small sample size, the confidence interval could not be calculated (point estimate: 0.26). Abbreviation: HTP, heated tobacco product.

The results of the subgroup analyses are presented in Fig. 2 and Table S1. AORs of calculated problem gambling were higher among women (exclusive combustible cigarette use, AOR = 5.91; 95% CI = 3.62–9.45, p < 0.001; exclusive HTP use, AOR = 8.65; 95% CI = 5.36–13.77, p < 0.001; and dual use, AOR = 25.13; 95% CI = 16.69–38.30, p < 0.001) than among men (exclusive combustible cigarette use, AOR = 2.51; 95% CI = 2.01–3.13, p < 0.001; exclusive HTP use, AOR = 1.55; 95% CI = 1.14–2.07, p = 0.004; and dual use, AOR = 8.70; 95% CI = 7.24–10.49, p < 0.001) for all the categories of tobacco product use. Regarding the age groups, the youngest age group (16–29 years) had the highest AORs of problem gambling for all the categories of tobacco product use (exclusive combustible cigarette use, AOR = 7.51; 95% CI = 4.67–12.06, p < 0.001; exclusive HTP use, AOR = 3.47; 95% CI = 1.91–6.12, p < 0.001; and dual use, AOR = 29.50; 95% CI = 20.76–42.65, p < 0.001). In addition, the AOR was significantly higher for individuals who used both combustible cigarettes and HTPs and were aged 66–83 years (AOR = 25.51; 95% CI = 11.57–57.98, p < 0.001).

## 4 Discussion

In this study, all smoking patterns, including the exclusive use of HTPs and dual use of HTPs and combustible cigarettes, were associated with problem gambling. Women had higher ORs for problem gambling associated with the use of each type of tobacco product than men did. Regarding age, the younger and older groups compared to that aged 45–65 years had higher ORs for problem gambling associated with the use of each type of tobacco product.

Previous studies have reported an association between problem gambling and nicotine dependence and combustible cigarette use or e-cigarette use in several countries. A *meta*-analysis of observational studies showed that 60.1% of individuals with problem gambling were nicotine-dependent (Lorains et al., 2011). An observational study in the UK suggested that individuals who used tobacco currently were over three times more likely than those who did not smoke to engage in problem gambling among the general adult population (n = 9003) (Griffiths et al., 2010). An online survey of university students in the US indicated that individuals who used e-cigarettes had a higher prevalence of gambling disorders than those who never used e-cigarettes (Grant et al., 2019). However, the relationship between HTPs—a relatively new form of tobacco—and problem gambling remains largely unexplored. In the present study, we examined whether exclusive HTP use and the dual use of HTPs and combustible cigarettes were associated with problem gambling. Although there were sex differences in smoking prevalence (Díaz et al., 2023, Taira et al., 2022), sex differences in the link between smoking and problem gambling were inconsistent in previous studies (Buja et al., 2017, Edgren et al., 2016). Therefore, we proposed the results of the sub-group analyses by sex. Moreover, while multiple studies have focused on adolescents (Buja et al., 2017, Edgren et al., 2016, Špolc et al., 2019), who are most vulnerable to smoking problems, few have assessed the heterogeneity of relationships with a wide age span sample, including middle-aged and older individuals. To compensate for this, we present the results of the sub-group analyses by age. This is the novelty and notability of our study, considering the limitations of previous research.

Neurobiological and psychological behavioural mechanisms may underlie the elevated prevalence of problem gambling among individuals who used combustible cigarettes. From a neurobiological standpoint, both nicotine and gambling activate mesolimbic dopamine pathways. Nicotine enhances dopamine release in the brain, thereby amplifying the sensation of reward, whereas the uncertain outcomes inherent in gambling similarly stimulate dopaminergic circuits (Bui et al., 2023, Di Chiara and Imperato, 1988, Limbrick-Oldfield et al., 2017, McGrath and Barrett, 2009, Pontieri et al., 1996, Zack and Poulos, 2004). This shared mechanism may result in reciprocal reinforcement, where nicotine potentiates the perceived reward value of gambling. From a psychological and behavioural perspective, individuals with high impulsivity are more prone to engage in both smoking and gambling (Weinberger et al., 2015). Moreover, symptoms of depression and anxiety have been shown to drive some individuals to smoke and gamble as mood regulatory strategies (Grant et al., 2009, Weinberger et al., 2015).

The high prevalence of problem gambling among individuals using HTPs may reflect the neurobiological effects of nicotine and use of HTPs for mood modulation. HTPs are known to induce nicotine dependence comparable to combustible cigarettes (Lau et al., 2022). Thus, HTPs may similarly “amplify” the reward value of gambling through dopaminergic activation. In addition IQOS, a common HTP, has been reported to provide distinct psychological rewards (Stone et al., 2022). This study and our results suggest that individuals seeking such rewards might be inclined to use HTPs and engage in problem gambling.

Individuals who used both cigarettes and HTPs showed a significantly higher AOR for problem gambling than those who did not smoke, which was notably higher than in other groups. Prior studies have indicated that individuals using multiple tobacco products have higher nicotine dependence than those using a single product (Huh et al., 2022). Furthermore, individuals using high-temperature HTP have been reported to have higher nicotine dependence than individuals who smoke cigarettes (Kitajima et al., 2025). Recently, nicotine dependence has been found to be strongly associated with gambling disorders (Dickerson et al., 2009). Based on these findings and the results of the present study, individuals who use both combustible cigarettes and HTPs have high nicotine dependence, which correlates with gambling addiction and explains the high rate of problem gambling among them.

This study had several limitations. First, because it employed a cross-sectional design, the temporal relationship between tobacco product use and problem gambling could not be definitively established. Therefore, a longitudinal study is necessary to examine causality. Second, our definition of “problem gambling” does not correspond to a clinically diagnosed gambling disorder as confirmed by psychiatrists. Instead, we assessed gambling behaviour using a questionnaire based on the SOGS and evaluated problem gambling using the PGSI. The association between tobacco use, including HTPs, and gambling disorder should be investigated in independent studies. Third, the online survey used may be subject to selection bias because our data were obtained via non-probability sampling between January 24 and February 27, 2024. This limits the generalisability of our findings (Cornesse et al., 2020). However, we applied IPW to adjust for a wide range of sociodemographic factors. Finally, our findings must be interpreted in light of the study setting. This study was conducted in Japan, and its external validity in countries with different social structures, populations, and ethnic compositions remains uncertain. Therefore, further research is needed to replicate and validate these results, both within Japan and internationally.

Despite these limitations, our study has several strengths. First, to the best of our knowledge, this study is the first to specifically examine the association between HTPs, a relatively new tobacco product, and problem gambling, and to further investigate the dual use of HTPs and combustible cigarettes. Conducting this survey in Japan, where HTP uptake has become substantial, allowed us to secure a sufficient number of individuals who used HTP for meaningful analysis. Second, we employed a multivariable logistic regression, adjusting for a comprehensive set of potential confounders, thereby isolating the independent association between tobacco product use and problem gambling. Finally, we performed stratified analyses by sex and age group to explore how these demographic factors modify the relationship between tobacco use, including HTPs, and problem gambling.

## 5 Conclusion

In this study, we examined whether combustible cigarettes, HTP, and the dual use of these products were associated with problem gambling. In particular, individuals who used both combustible cigarettes and HTPs had a higher prevalence of problem gambling. The results of this study may inform both policymaking and clinical practice, if future studies establish a causal relationship between tobacco use and problem gambling. For policymakers, combining gambling-related interventions with smoking cessation measures, such as implementing smoke-free policies in gambling venues, may be an effective approach. For clinicians, these associations may support the prevention and treatment of problem gambling, for example by screening smokers, particularly dual users, for problematic gambling behaviors. Moreover, even based solely on the present findings, smoking, including heated tobacco products, may represent an important factor to consider in policy development and clinical prevention strategies for problem gambling. To further examine this association, longitudinal studies are necessary to clarify the causal relationship, in addition to surveys of other populations and countries.

## Role of funding sources

This study was supported by the Health Labor Sciences Research Grants (22FA1002, 22FA1010, 22FA2001, 23FA1004, 23JA1003, and 23EA1009), the 10.13039/501100001691Japan Society for the Promotion of Science (JSPS) KAKENHI Grants (20H00040 and 21H03203), and the intramural fund of the National Institute for Environmental Studies. The funders had no role in the design and conduct of the study; collection, management, analysis, and interpretation of the data; preparation, review, or approval of the manuscript; and decision to submit the manuscript for publication.

## CRediT authorship contribution statement

**Minami Takeda:** Writing – original draft, Visualization, Software, Project administration, Formal analysis, Data curation, Conceptualization. **Takashi Yoshioka:** Writing – review & editing, Visualization, Resources, Project administration, Methodology, Investigation, Conceptualization. **Kosuke Shido:** Writing – review & editing, Validation, Software, Resources, Methodology. **Atsushi Hozawa:** Writing – review & editing, Validation. **Takahiro Tabuchi:** Writing – review & editing, Validation, Resources, Project administration, Investigation, Funding acquisition, Conceptualization.

## Declaration of competing interest

The authors declare the following financial interests/personal relationships which may be considered as potential competing interests: TY received the Japan Society for the Promotion of Science (JSPS) KAKENHI Grants (grant number: 25K02856) for research aimed at reducing the number of potential addiction patients. TT received financial support including consulting and lecture fees, from Daiichi Sankyo Healthcare Co., Ltd., Data Seed Inc., Workout-Plus LLC (in the last 36 months), Johnson & Johnson K.K., and EMMA Co., Ltd outside this work. TT also received grants from the Ministry of Health Labour and Welfare (grant numbers: 23FA1004 and 23EA1001), and the JSPS (grant numbers: 25H01079, 24H00663, 23H03160), all of which are regarding tobacco-control policy research. MT, KS, and AH have no conflict of interest to declare.

## Data Availability

The authors do not have permission to share data.
