# Supplementary material for: Association between tobacco use, including heated tobacco products, and problem gambling: A cross-sectional study
Source: Addict Behav Rep. 2026 Feb 21;23:100678. doi: 10.1016/j.abrep.2026.100678 (PMC12950345; doi:10.1016/j.abrep.2026.100678)
Supplement: Supplementary Data 1 [file mmc1.docx]

**Supplementary material**

**Table S1** Full estimates of the subgroup analysis

|  | n | Problem gambling, weighted % | AOR (95% CI) | p-value |
| --- | --- | --- | --- | --- |
| **Male** |  |  |  |  |
| Non-current/never use | 4732 | 6.6 | 1.00 (ref.) | (ref.) |
| Exclusive cigarette use | 1192 | 13.5 | 2.51 (2.01–3.13) | <0.001 |
| Exclusive HTP use | 566 | 13.0 | 1.55 (1.14–2.07) | 0.004 |
| Dual use | 1159 | 42.4 | 8.70 (7.24–10.49) | <0.001 |
| **Female** |  |  |  |  |
| Non-current/never use | 3707 | 2.1 | 1.00 (ref.) | (ref.) |
| Exclusive cigarette use | 358 | 14.8 | 5.91 (3.62–9.45) | <0.001 |
| Exclusive HTP use | 203 | 22.2 | 8.65 (5.36–13.77) | <0.001 |
| Dual use | 305 | 39.9 | 25.13 (16.69–38.30) | <0.001 |
| **16–29 years** |  |  |  |  |
| Non-current/never use | 1459 | 6.9 | 1.00 (ref.) | (ref.) |
| Exclusive cigarette use | 166 | 35.6 | 7.51 (4.67–12.06) | <0.001 |
| Exclusive HTP use | 141 | 31.2 | 3.47 (1.91–6.12) | <0.001 |
| Dual use | 505 | 64.5 | 29.50 (20.76–42.65) | <0.001 |
| **30–45 years** |  |  |  |  |
| Non-current/never use | 2263 | 6.7 | 1.00 (ref.) | (ref.) |
| Exclusive cigarette use | 362 | 21.3 | 2.34 (1.68–3.25) | <0.001 |
| Exclusive HTP use | 237 | 18.9 | 2.54 (1.77–3.62) | <0.001 |
| Dual use | 484 | 38.6 | 6.72 (5.15–8.80) | <0.001 |
| **46–65 years** |  |  |  |  |
| Non-current/never use | 2793 | 4.0 | 1.00 (ref.) | (ref.) |
| Exclusive cigarette use | 657 | 9.0 | 1.81 (1.27–2.58) | 0.001 |
| Exclusive HTP use | 331 | 6.9 | 1.21 (0.72–1.94) | 0.4 |
| Dual use | 382 | 25.4 | 5.88 (4.18–8.21) | <0.001 |
| **66–83 years** |  |  |  |  |
| Non-current/never use | 1924 | 1.5 | 1.00 (ref.) | (ref.) |
| Exclusive cigarette use | 365 | 5.5 | 3.69 (1.86–7.10) | <0.001 |
| Exclusive HTP use | 60 | 0.3 | 0.26 (<4.98) | 0.6 |
| Dual use | 93 | 21.1 | 25.51 (11.57–57.98) | <0.001 |

**Abbreviations:** AOR, adjusted odds ratio; HTP, heated tobacco products 95% CI, 95% confidence interval.

**Note.** Due to small sample size, the confidence interval could not be calculated (point estimate: 0.26).
